# J. Bradfield

**Published:** 1882-01-20

**Authors:** 


J. BRADFIELD, Druggist, Atlanta. See the advertisement of this house, in our Journal, which makes a specialty of Physicians' Supplies. Dr. Bradfield's long connection with the drug business, and his extensive acquaintance throughout the Southern States, his accommodating disposition and his great energy as a business man, will doubtless secure for his house a liberal share of the patronage in his line.

